# Outcome of limb lengthening as a treatment for shortening following successful replantation of traumatic leg amputation: experience with 21 patients

**DOI:** 10.3389/fsurg.2025.1686865

**Published:** 2025-10-03

**Authors:** Caifeng Wu, Kai Liu, Shengquan Ren, Mingming Liu, Xiaoheng Ding, Aihemaitijiang Yusufu

**Affiliations:** 1 Department of Trauma and Microreconstructive Surgery, The First Affiliated Hospital of Xinjiang Medical University, Urumqi, Xinjiang, China; 2 Department of Orthopaedics, The Affiliated Hospital of Southwest Medical University, Luzhou, Sichuan, China; 3 Department of Hand and Foot Microsurgery, Qingdao University Affiliated Hospital, Qingdao, Shandong, China; 4 Department of Hand Surgery, No. 971 Hospital of the Chinese PLA Navy, Qingdao, Shandong, China

**Keywords:** external fixation, ilizarov technique, limb lengthening, lower limb, shortening deformity

## Abstract

**Objective:**

The purpose of this study was to evaluate the outcomes of limb lengthening based on the Ilizarov technique in the treatment of limb shortening following successful replantation of traumatic lower leg amputation.

**Methods:**

The clinical records and consecutive x-ray photographs of patients with limb shortening deformities following successful replantation of traumatic lower leg amputation treated by limb lengthening using an external fixator were analyzed retrospectively, from January 2012 to December 2022. The demographic data, initial injury, previous treatment, and postoperative data were collected. Paley classification was applied to assess the bone and functional outcomes. The lower extremity functional scale (LEFS), visual analog scale (VAS), and 36-item Short Form Health Survey of life quality (SF-36) were used to evaluate and compare the results of the affected limbs.

**Results:**

A total of 21 patients with a mean age of 42.71 ± 7.96 years, consisting of 17 males (80.9%) and 4 females (19.1%), were successfully treated by limb lengthening using an external fixator. The mean length of limb shortening after limb replantation is 9.93 ± 2.88 cm. The mean external fixation time of this cohort was 16 ± 5.27 months, with a mean external fixation index of 1.59 ± 0.14 month/cm. In bone results, there were 14 cases in excellent, and 6 cases in good, with an excellent and good rate of 95.2%. In functional results, there were 15 cases of excellent, and 5 cases of good, with an excellent and good rate of 95.2%. The knee joint displayed an average over-extension range of motion of 2.9 ± 0.85° (0°–5°), with an average flexion range of 114.7 ± 3.05° (105°–140°). Among the patients, except for the 2 cases of tenodesis, the remaining individuals exhibited an average plantar flexion of 23.2 ± 3.34° (10°–40°) and an average dorsiflexion of 15.1 ± 3.44° (10°–25°). The outcomes of plantar sensation recovery were as follows: 6 cases were classified as S3_+_, 11 cases as S3, and 4 cases as S2. The LEFS and SF-36 scores followed a characteristic “V”-shaped trajectory, initially declining before subsequently increasing (*P* < 0.05). VAS scores generally showed a trend opposite to that of the LEFS functional scores (*P* < 0.05).

**Conclusion:**

Limb lengthening based on the Ilizarov technique was a safe and effective method for treating shortening following successful replantation of traumatic lower leg amputation, and it could yield satisfactory postoperative bone and functional results. The long treatment period usually associated with this method increases the risk of complications, necessitating good patient compliance with meticulous postoperative management and follow-up guidance to minimize these risks.

## Introduction

With the advancement in industry, agriculture, and transportation, high-energy trauma frequently results in comminuted fractures combined with extensive soft tissue injuries and severe limb amputation (1–4). Achieving effective limb salvage remains a huge challenge for orthopaedic surgeons when treating these severe limb injuries since the involvement of extensive damage to bones, blood vessels, nerves, and tendons. In some cases, the trauma itself may have already resulted in limb shortening deformity.

Advancements in microsurgical techniques have facilitated effective replantation and limb salvage through radical debridement and direct anastomosis of blood vessels, nerves, and tendons (5). However, achieving complete reconstruction of the original limb length remains challenging in cases of comminuted fractures that cannot be fully reduced, or when blood vessels, nerves, and tendons are severely damaged or have defects. In such instances, acute shortening may be necessary to ensure successful limb salvage. While one-stage free tissue flap grafting can address soft tissue defects and help maintain limb length, this complex surgical procedure requires interdisciplinary collaboration and extensive support from multiple surgical teams (6). In recent decades, the Ilizarov technique has emerged as an effective method for the treatment of bone defects, bone deformities, and limb length discrepancy (7–10). However, there are few clinical reports on the application of limb lengthening as a treatment of shortening following successful replantation of traumatic lower leg amputation.

Therefore, the purpose of this study was to evaluate the outcomes of limb lengthening based on the Ilizarov technique in the treatment of limb shortening following successful replantation of traumatic lower leg amputation.

## Materials and methods

Permission from the Ethics Committee was obtained, and informed consent was received from all patients. The clinical records and consecutive x-ray photographs of patients with limb shortening deformities following successful replantation of traumatic lower leg amputation treated with limb lengthening using an external fixator were analyzed retrospectively from January 2012 to December 2022. Limb shortening deformities occurred to enable successful replantation after effective debridement, necessitated by extensive damage to the bones, blood vessels, nerves, and tendons.

### Inclusion and exclusion criteria

Inclusion criteria included: patients with a limb shortening of >3 cm following successful replantation of traumatic lower leg amputation, a strong willingness to restore limb length, and treated by limb lengthening using external fixation. Besides, all patients underwent a comprehensive preoperative assessment. Bone union at the replantation site was confirmed using plain radiographs before initiating limb lengthening surgery. Soft tissues were evaluated for both integrity and vascular status to exclude active infection or ischemia. We included only those patients with confirmed fracture union, stable soft tissues, and no evidence of active infection or ischemic compromise. Patients with severe cardiovascular comorbidities, incomplete medical records, poor compliance, and follow-up time <20 months were excluded.

The demographic data, initial injury, and previous treatment were documented. Muscle or tendon injuries typically require approximately 6 weeks after suturing to heal completely, while nerve tissue suturing usually heals in approximately 3–4 weeks (11). Additionally, the morphology and structure of elastic tissue at the anastomotic site generally return to a normal state, 30 days after vascular anastomosis. Therefore, the limb lengthening procedures were co nducted at least 6 weeks after the successful limb replantation to ensure a successful outcome (11).

### Patients’ data

**Figure 1:**
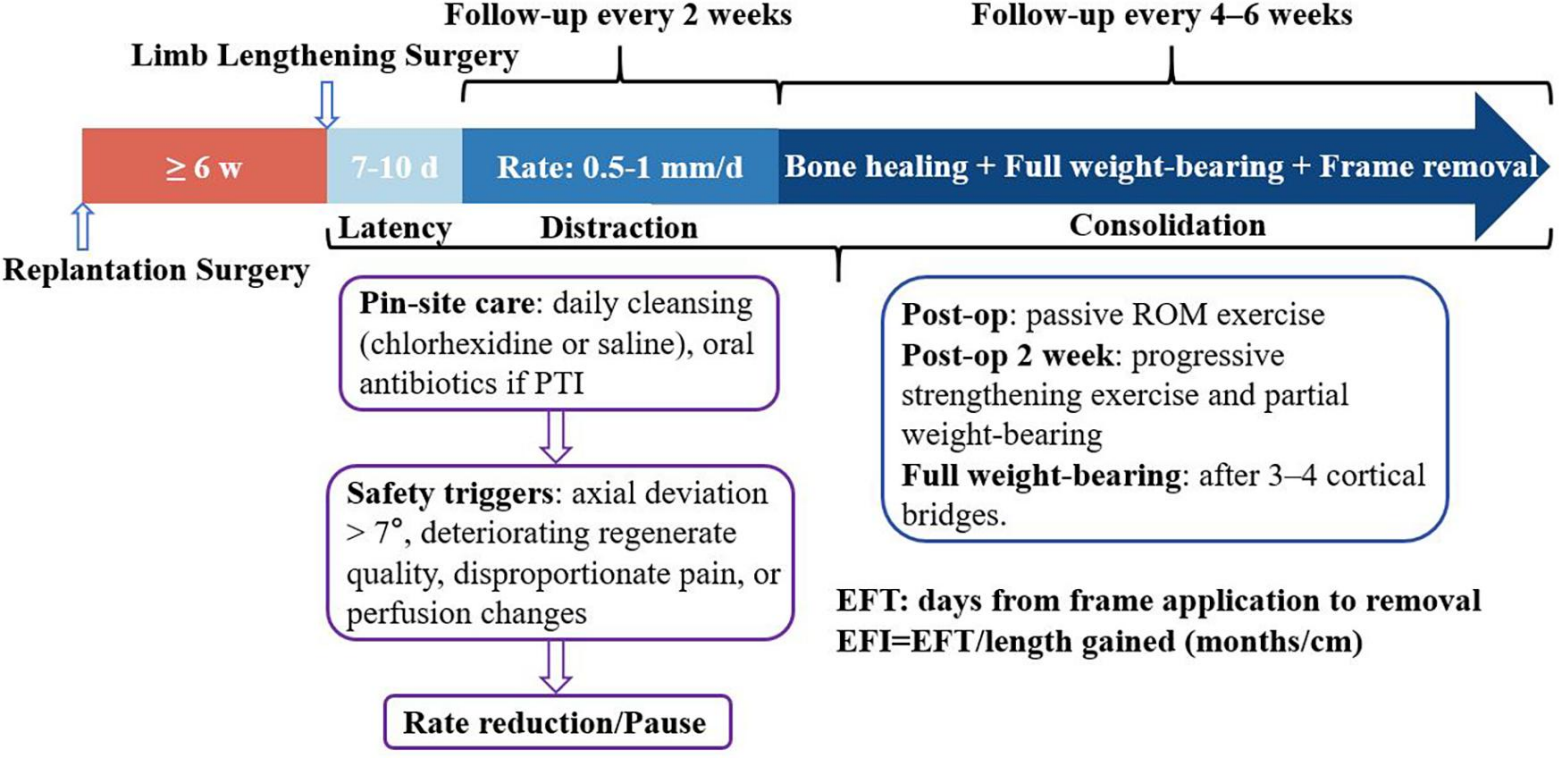
Treatment protocol.

A total of 21 patients with a mean age of 42.71 ± 7.96 years, consisting of 17 males (80.9%) and 4 females (19.1%). The mean postoperative follow-up time was 27 ± 4.01 months. The mechanisms of injury were all motor vehicle traffic accidents, with 2 cases accompanied by open fractures of the contralateral tibia and fibula (Gustilo-Anderson type II). Twenty-one lower legs underwent acute shortening to facilitate successful replantation following effective debridement due to extensive damage to the bones, blood vessels, nerves, and tendons. Among the locations of limb replantation in patients, there were 13 cases of the distal tibia, 6 cases of the mid tibia, and 2 cases of the proximal tibia. In 19 cases, the end-to-end anastomosis of anterior and posterior tibial vessels was successfully performed. The free anterolateral thigh flap was utilized to bridge the anterior tibial vessels and repair tissue defects in 2 cases, including tendons and bone exposure. The nerves (tibial nerve and deep peroneal nerve) and tendons were repaired without tension. The mean time to replantation surgery was 3.59 ± 0.8 hours, and all were fixed with simple external fixators. Primary wound healing was observed in 19 patients (90.4%), and soft tissue defects occurred in 2 patients (9.6%). The mean delay time in lengthening was 88.04 ± 67.8 days. The mean length of limb shortening after limb replantation is 9.93 ± 2.88 cm (Table 2). The treatment timeline was presented in Figure 1.

**Table 2:** Baseline data of 21 patients.

Number	Gender	Age	Side/type of injury	Time to replantation (hours)	Delay of lengthening (day)	Amount of limb lengthening (cm)
1	M	36	R/TA	5.0	73	10.3
2	M	51	L/TA	5.0	360	14.1
3	M	60	R/TA	4.0	61	8.8
4	M	49	R/TA	3.5	56	10.0
5	M	51	L/TA	3.0	73	6.9
6	M	30	R/TA	4.0	75	5.9
7	M	44	R/TA	4.0	68	6.8
8	M	42	R/TA	4.0	71	9.9
9	M	52	R/TA	3.0	92	10.0
10	F	39	L/TA	3.0	63	9.2
11	M	37	L/TA	4.5	67	10.8
12	F	38	R/TA	4.0	70	10.5
13	M	37	R/TA	3.0	57	11.5
14	F	47	R/TA	5.0	77	12.0
15	M	42	L/TA	3.5	62	9.6
16	M	43	R/TA	2.0	60	8.6
17	M	47	R/TA	4.0	185	10.0
18	M	27	R/TA	5.0	63	5.4
19	F	36	L/TA	4.0	66	12.3
20	M	50	R/TA	4.5	82	12.0
21	M	39	R/TA	4.0	68	6.2

F, female; L, left; M, Male; R, right; TA, traumatic amputation.

### Surgical technique

The detailed preoperative plan was first conducted by our experienced surgeon using radiographs, CT scans, and three-dimensional reconstructed images. After general anesthesia, the patient was supinely positioned on the operating bed. The length of the affected limb was measured and marked on the medial side, and the insertion points for the external fixator and the anticipated positions of each ring were determined. Typically, two full rings were installed at the proximal tibia, one full ring at the middle tibia, two full rings at the distal end of the tibial fracture line, and one U-ring at the foot. It was crucial to ensure that the rings on the lower limbs were parallel to each other. An intraoperative x-ray was then used to confirm that the axis of the external fixator was paralleled to the tibial axis and that the motion axis was aligned with the ankle's flexion and extension axis.

The proximal rings were positioned in the superior and inferior planes relative to the tibial tubercle, and the distal rings were placed as close to the articular surface of the distal tibia as possible. Each ring was secured with a 2 mm diameter cross Kirschner wire, and the U-ring on the foot was stabilized with a cross Kirschner wire at the heel and a Kirschner wire at the forefoot, crossing the first to fifth metatarsals. All K-wires were tightened using a wire tensioner. A connecting rod was installed distal to the U-ring of the foot to lengthen the Achilles tendon and prevent foot drop deformity.

The osteotomy was performed approximately 4–5 cm below the tibial tubercle. A longitudinal incision of about 5 cm was made along the outer edge of the tibia centred on the osteotomy plane, and the tibia was cut with an osteotome following intermittent drilling with Kirschner wires. Using the same osteotomy method, a 3 cm incision was made laterally to the middle fibula to expose and cut the fibula. After the x-ray confirmed the proper alignment of the osteotomy line, each connecting nut was tightened.

### Postoperative management

The distraction phase commenced after a latency of 7–10 days, progressing at a rate of 1 mm per day, and was completed 2–4 times. Patients were advised to avoid weight-bearing activities on the second postoperative day (12). After that, they were encouraged to walk with partial weight bearing during the distraction and consolidation phases. Full weight-bearing is initiated after 3–4 cortical bridges. The process of bone regeneration in the distraction area was radiographically monitored every two weeks during the distraction phase and monthly during the consolidation phase.

Patients received instructions on pin tract care to prevent infection. The blood circulation and clinical symptoms of the affected limb were also monitored. The distraction rate was reduced to 0.25–0.5 mm per day, completing the process 4–6 times, when symptoms such as pain, swelling, or severe skin irritation occurred in the affected limb. Painkillers [non-steroidal anti-inflammatory drugs (NSAIDs)] and neuromodulators were employed for the short-term management of temporary pain resulting from the distraction process. These medications were gradually discontinued as the pain subsided. Simultaneously, interventions such as dressing changes and physiotherapy were conducted to maintain cleanliness around the pin tract and alleviate longitudinal skin tension. After the termination of the distraction phase, the external frame could be removed once two-thirds of the rounded cortical bone was visible in the distraction area. Following the removal of the external fixator, patients were advised to avoid weight-bearing walking for two weeks, instead utilizing a brace or crutches. After that, full weight-bearing walking was encouraged.

### Data collection and outcome evaluation

The postoperative data were collected, including delay in lengthening (days), amount of limb lengthening (cm), bone union time (BUT, months), external fixation time (EFT, months), external fixation index (EFI, months/cm), complications, and additional procedures. After discharge, patients were followed at 1, 3, 6, 9, 12, 18, and 24 months. Paley classification (13) was applied to assess the bone and functional outcomes and postoperative complications (Table 1). Physical examinations included evaluation of knee and ankle range of motion (ROM), and soft tissue conditions were recorded. The plantar sensation function of the affected limbs after replantation and limb lengthening was evaluated using the British Medical Research Council (BMRC) grading system (14). Besides, the lower extremity functional scale (LEFS), visual analog scale (VAS), and 36-item Short Form Health Survey of life quality (SF-36) were used to evaluate and compare the results of the affected limbs at pre-operative limb lengthening surgery, end of distraction, consolidation 3 months, consolidation 6 months, and final follow-up.

**Table 1:** Outcome definitions.

Parameter	Definition
BUT-replantation site	Date of first imaging meeting bridging of ≥3 of 4 cortices on orthogonal radiographs; CT obtained when radiographs were equivocal (slice thickness/window documented).
BUT-lengthening regenerate	Same radiographic/CT criteria, recorded separately.
EFT	Duration from frame application to removal.
EFI	EFT/length gained (months/cm).
Delay of lengthening	The interval between replantation surgery and limb lengthening surgery.
Complication	Complication include any local or systemic intraoperative or perioperative complication, difficulty during distraction or fixation that remains unsolved at the end of treatment period, and any early or late post-treatment difficulty.

Two blinded readers independently assessed imaging; disagreements were resolved by consensus/third reader.

### Statistical analysis

The categorical variables were entered into a Microsoft Excel spreadsheet, presented as frequency and percentage, and then analyzed using the GraphPad Prism v10.0 (San Diego, CA, US). Gaussian distribution was assessed via the Shapiro–Wilk test, while variance homogeneity was evaluated through Levene's method. Where parametric conditions were satisfied, inter-group comparisons between two cohorts employed Student's *t*-test, whereas multi-group analyses utilized one-way ANOVA with Tukey *post-hoc* adjustments. For non-normally distributed datasets or heteroscedastic variances, non-parametric alternatives were implemented: the Mann–Whitney *U*-test for pairwise analyses and the Kruskal–Wallis H test with Dunn's correction for multi-variable comparisons. *P* < 0.05 was considered to indicate a statistical difference.

## Results

Twenty-one patients (100%) with limb shortening deformity after replantation of lower leg amputation were successfully treated by limb lengthening using a circular external fixator, with a mean bone union time of 14.24 ± 4.76 months. The mean external fixation time of this cohort was 16 ± 5.27 months, with a mean external fixation index of 1.59 ± 0.14 month/cm (Table 3). At the last follow-up, Paley's classification was used to evaluate the bone and functional outcomes. In bone results, there were 14 cases in excellent, and 6 cases in good, with an excellent and good rate of 95.2%. In functional results, there were 15 cases of excellent, and 5 cases of good, with an excellent and good rate of 95.2%.

**Table 3:** Clinical data of 21 patients.

Number	EFT (month)	EFI (month/cm)	BUT- lengthening regenerate (month)	Complication	Additional procedure	Outcome	Follow-up time (month)
1	15.1	1.47	14.5	Pain	Oral NSAIDs	Union	29.7
2	22.9	1.53	21.0	Pain	Oral NSAIDs	Union	22.9
3	14.6	1.66	13.0	–	–	Union	22.0
4	20.0	1.68	19.0	Foot valgus deformity	Tenodesis	Union	25.8
5	11.2	1.63	10.0	–	–	Union	32.8
6	9.2	1.56	8.3	–	–	Union	25.4
7	9.8	1.45	9.0	–	–	Union	29.4
8	14.5	1.47	13.0	SN, pain	Debridement, Oral NSAIDs	Union	29.9
9	16.4	1.64	14.5	PTI	Dressing change + oral antibiotics	Union	32.1
10	14.2	1.55	13.6	Delay union in the distraction area	ABG	Union	20.6
11	19.0	1.76	17.0	PTI	Dressing change	Union	27.3
12	15.2	1.45	13.0	–	–	Union	24.5
13	20.0	1.74	18.2	Foot valgus deformity, pain	Tenodesis, Oral NSAIDs	Union	28.8
14	20.2	1.68	16.1	–	–	Union	24.7
15	18.9	1.97	18.3	–	–	Union	22.8
16	13.3	1.55	11.0	–	–	Union	34.1
17	16.8	1.68	13.4	PTI	Dressing change	Union	31.0
18	7.2	1.35	6.6	–	–	Union	32.3
19	20.5	1.67	19.0	PTI, pain	Dressing change, Oral NSAIDs	Union	24.7
20	20.4	1.70	16.2	Pain	Oral NSAIDs	Union	22.7
21	8.3	1.34	7.5	–	–	Union	25.0

ABG, autogenous bone grafting; BUT, bone union time; EFI, external fixation index; EFT, external fixation time; NSAIDs, Non-steroidal anti-inflammatory drugs; PTI, pin tract infection; SN, skin necrosis.

Before limb lengthening, the knee joint showed an average over-extension ROM of 3.1 ± 1.04° (0°–5°), with an average flexion range of 110.5 ± 3.61° (100°–135°). Except for the 4 cases that underwent ankle joint fusion caused by initial trauma, ROM of the ankle joint of the remaining patients showed an average plantar flexion of 25.3 ± 2.84° (10°–40°) and an average dorsiflexion of 16.4 ± 2.85° (10°–20°). The recovery of plantar sensation was assessed according to the BMRC grading system, resulting in 3 cases classified as S3_+_, 10 cases as S3, and 8 cases as S2. At the final follow-up, the knee joint showed an average over-extension range of motion of 2.9 ± 0.85° (0°–5°), with an average flexion range of 114.7 ± 3.05° (105°–140°). Among the patients, except for the 4 cases of ankle joint fusion, the remaining individuals exhibited an average plantar flexion of 23.2 ± 3.34° (10°–40°) and an average dorsiflexion of 15.1 ± 3.44° (10°–25°). The outcomes of plantar sensation recovery were as follows: 6 cases were classified as S3_+_, 11 cases as S3, and 4 cases as S2.

**Figure 2:**
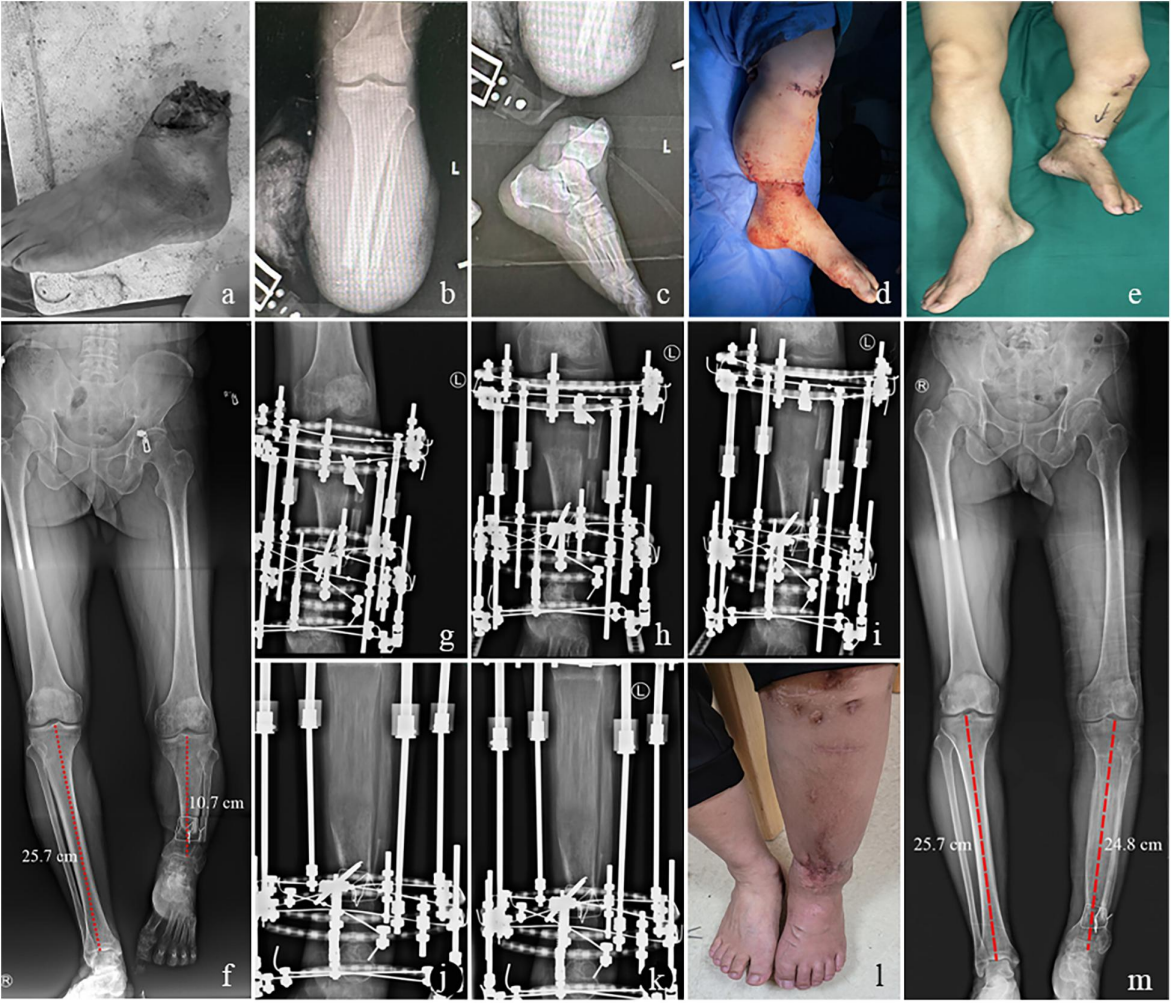
A 51-year-old male with limb shortening deformity after replantation of lower leg amputation treated by limb lengthening using an external fixator. **(a)** Traumatic amputation of the distal left lower leg. **(b,c)** x-rays of left tibia. **(d)** Replantation of the affected limb after shortening approximately 15 cm. **(e)** Successful limb salvage. **(f)** The full-length x-ray of the lower limb showed a shortening of about 10 cm in the left lower leg. (g) Postoperative x-ray of the left tibia. **(h–j)** x-ray of the left tibia at one month, 3 months and 5 months after surgery. **(k,l)** The consolidation phase was completed with satisfactory bone union and limb function recovery at the 21 postoperative month. **(m)** The full-length x-ray of the lower limb revealed a shortening of approximately 0.9 cm in the left lower leg, which is generally considered acceptable.

**Figure 3:**
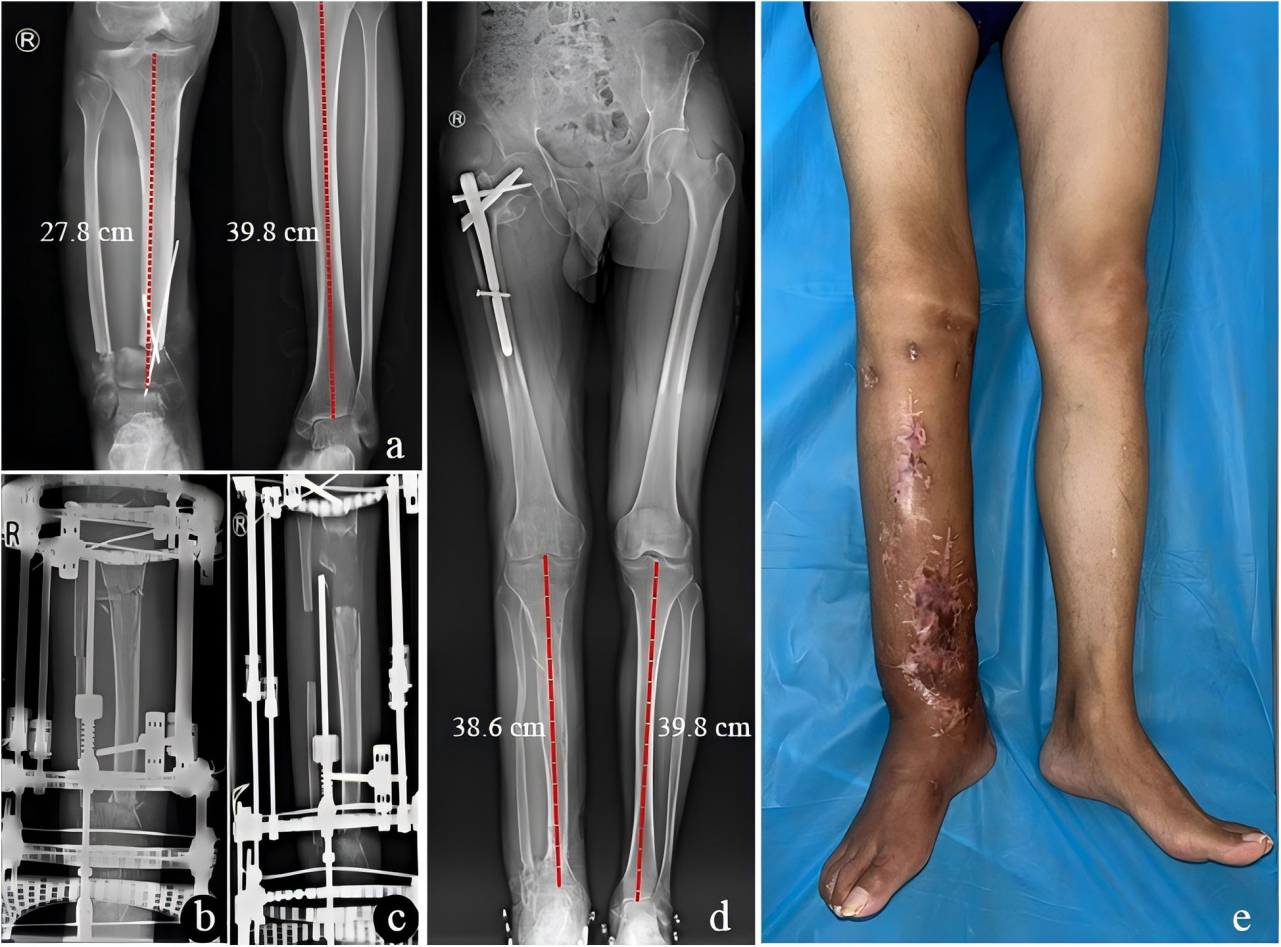
A 37-year-old male with limb shortening deformity after replantation of lower leg amputation treated by limb lengthening. **(a)** Successful limb salvage and replantation of the affected limb after shortening approximately 12 cm. **(b)** x-ray of the right tibia before limb lengthening. **(c)** Three months after limb lengthening. **(d,e)** The implantation of the Proximal Femur Bionic Nail (PFBN) in the proximal femur for initial intertrochanteric fracture may not affect the overall study findings, as it had no impact on the primary endpoint of limb length discrepancy correction or the functional outcomes based on tibial measurements. The consolidation phase was completed with satisfactory bone and functional outcomes after 19 postoperative months.

Complications were observed in 8 patients (38.1%). Pin tract infections in 4 patients (19%) were managed with dressing changes and oral antibiotics. Six patients (28.5%) with pain in the affected limbs were effectively treated by oral NSAIDs. Skin necrosis in one patient (4.7%) was treated by debridement. Two cases (9.5%) experienced foot valgus deformity and were treated by tenodesis for ankle stability and supramalleolar osteotomy for valgus deformity. The delayed union of the distraction area in one patient (4.7%) was successfully managed by autogenous bone grafting. The typical cases were shown in Figures 2, 3.

**Figure 4:**
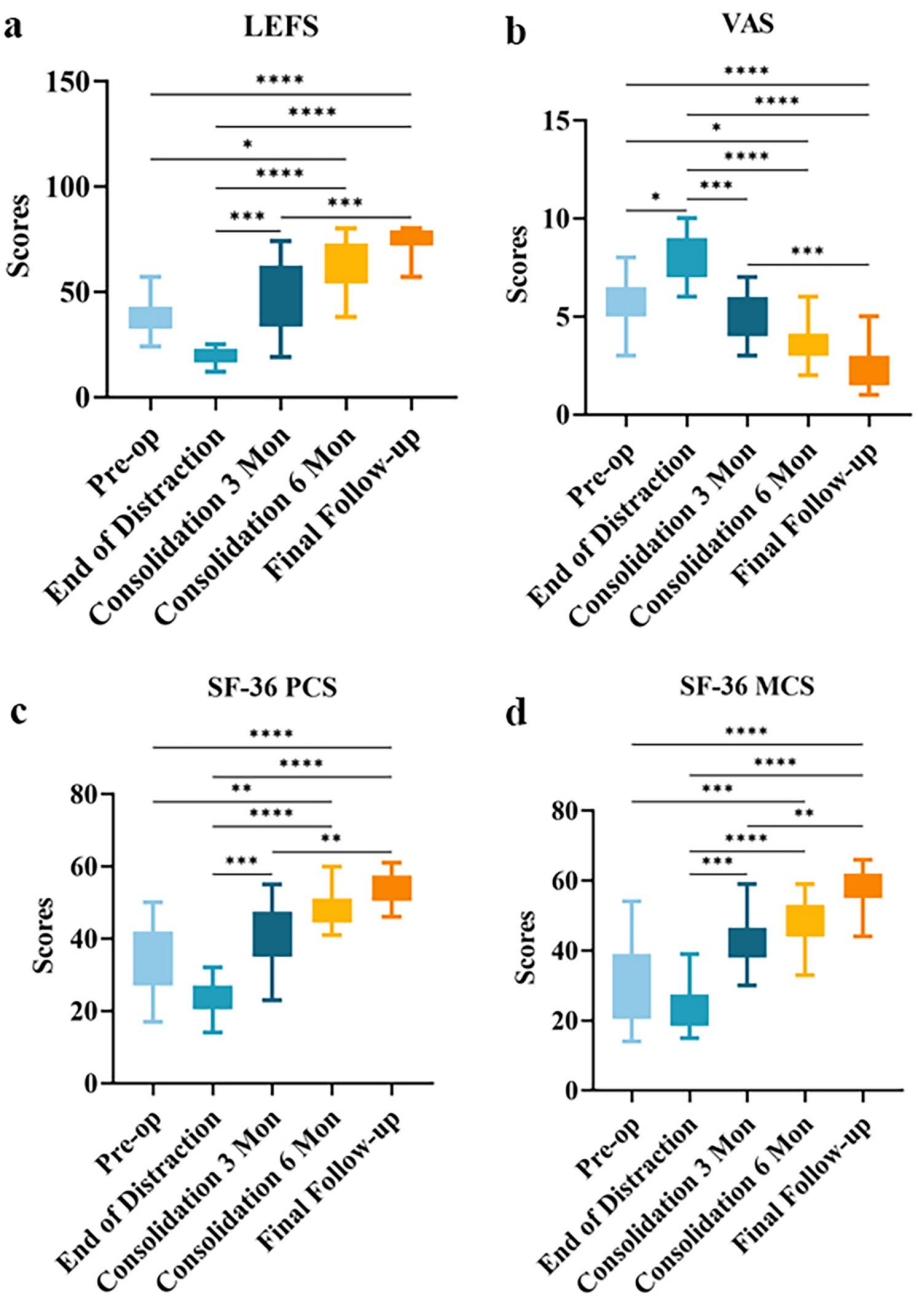
Comparison of limb function results before and after limb lengthening surgery for patients. **(a)** LEFS. **(b)** VAS. **(c)** SF-36 PCS. **(d)** SF-36 MCS. (* *P* < 0.05, ** *P* < 0.01, *** *P* < 0.001, **** *P* < 0.0001).

The LEFS and SF-36 scores followed a characteristic “V”-shaped trajectory, initially declining before subsequently increasing (Figure 4, *P* < 0.05). VAS scores generally showed a trend opposite to that of the LEFS functional scores. This pattern indicated that a temporary functional decline during the distraction phase represented a normal part of the process (*P* < 0.05). Pain served as the primary barrier to functional recovery, making effective pain management a prerequisite for rehabilitation. A marked rebound in LEFS and SF-36 scores occurred during the 3-month consolidation phase (*P* < 0.05). The Mental Component Summary (MCS) of the SF-36 typically recovered more slowly than the Physical Component Summary (PCS), reaching its lowest point at the end of distraction. These findings underscore that psychological support is as crucial as physical treatment throughout the therapy. By the final follow-up, most patients had achieved full weight-bearing. Continued rehabilitation training significantly improved muscle strength and joint range of motion, enabling patients to resume many daily activities, such as prolonged walking and stair climbing. These results affirm the effectiveness of limb lengthening in correcting post-traumatic limb shortening deformities.

## Discussion

Limb lengthening based on the Ilizarov technique can effectively address the shortening deformities following limb replantation caused by high-energy injuries. Although the limb lengthening process is similar to bone transport in terms of proximal tibial osteotomy level, osteotomy method selection, bone regeneration, and timing of external fixator removal, there are specific considerations regarding the timing of limb lengthening (8, 15). Besides, the greater the extent of limb shortening, the more challenging the treatment becomes, encompassing aspects such as the timing of limb lengthening, patient compliance, the risks and management of postoperative complications, and the evaluation of both bone and functional outcomes.

Limb lengthening differs from bone transport and limb lengthening used for congenital malformations primarily in that it involves lengthening the limb while also promoting self-repair and lengthening of soft tissues, including nerves, blood vessels, and tendons during replantation (16). Consequently, limb lengthening should only be performed after the soft tissues have achieved a nearly normal physiological structure. Qiu et al. (11) have shown that anastomosed nerves take approximately 4 weeks to develop axons through the anastomosis. Although vascular anastomosis repairs the intima with neo-endothelium within one postoperative week, the local morphological structure takes around 4 weeks to approach normalcy. However, complete regeneration of the injured muscles is challenging, with desmoplasia and scar repair usually being substantially complete by 8 weeks. For the physical structure of the common injured level of the lower leg, which often involves muscle and tendon tissues, complete healing typically requires 6 weeks (11). Therefore, limb lengthening should ideally be performed at least 8 weeks after successful replantation of the severed limb. In this study, the delay lengthening time of patients who opted for limb lengthening surgery was between 8 and 52 weeks after successful replantation and received satisfactory bone and functional outcomes, which reflects careful consideration of these factors. Besides, in this study, amputations occurred at various tibial levels: 13 were distal, 6 were mid-tibial, and 2 were proximal. We considered that proximal amputations, being closer to the knee, likely have greater consequences for muscle function and neurovascular integrity. In contrast, distal tibial amputations may better preserve functional capacity and biomechanics of the lower limb.

The rate of limb lengthening not only impacts bone healing but also significantly affects soft tissues (17). While there is no definitive conclusion on the safe limitation for nerve lengthening, it is known that the maximum length of nerve lengthening is related to the rate of lengthening (18). Nerves normally tolerate a slower lengthening rate better than a rapid rate. At a lengthening rate of 1 mm/day, peripheral nerves can be safely and effectively lengthened without obvious histological damage (16, 17). However, lengthening at 2 mm/day may lead to abnormal nerve structures and circulation compromise, resulting in irreversible damage such as Wallerian degeneration of the nerve (19). Additionally, the risk of adverse effects on soft tissue can be reduced by maintaining a rate of 1 mm/day while increasing the frequency of lengthening (4). For instance, limb lengthening divided into 4–6 times within the rate of 1 mm/day may preserve neural structure almost completely. Rapid lengthening may also adversely affect blood vessels. Xu et al. suggested that lengthening at a rate of 1 mm/day (4–6 times) may not harm blood vessels, however, it can enhance the regeneration of neovascular branching in the lengthening area, slightly improving the circulation of regenerated tissue (20). For skin and tendon tissues, mechanical stress promotes the proliferation and differentiation of skin cells. Studies indicated that muscle or tendon tissues showed no significant damage when lengthened at 1 mm/day to 50% of the length of adjacent long bones (21, 22). However, progressive muscle tissue damage may result as the extension amount increases when the lengthening rate ≥1.5 mm/day. In our cohort, the distraction rate was 1 mm/day, divided into 2–4 times. However, in clinical practice, it may be challenging to maintain a consistent distraction rate, particularly in the later phase of limb lengthening. As the distraction progresses, patient discomfort (potential pain) tends to increase, necessitating a reduction in the distraction rate. Nonetheless, a slower distraction rate may lead to premature healing of the regenerated bone, complicating the lengthening process. Therefore, if the patient cannot tolerate the initial distraction rate, it is advisable to reduce the extension rate to 0.5 mm/day or even 0.25 mm/day, divided into 2–4 times. It is generally not recommended to pause the distraction phase.

Effective pain management and rational use of neuromodulators during limb lengthening can not only improve patient comfort but also enhance overall treatment outcomes by promoting adherence to rehabilitation plans and reducing the risk of complications (14, 23, 24). The prolonged use of nonsteroidal anti-inflammatory drugs may hinder bone formation and decelerate the rate of bone healing due to their anti-inflammatory properties. However, short-term usage is generally regarded as safe (25). In this cohort, six patients (28.5%) with pain in the affected limbs were effectively treated by temporary oral NSAIDs. Therefore, it is recommended to carefully monitor the duration and dosage of NSAIDs to strike a balance between pain relief and bone healing. Although certain analgesics and neuromodulators may exert indirect effects on bone healing, meticulous management and a multidisciplinary approach may effectively mitigate these impacts, ensuring successful pain treatment while fostering optimal bone recovery. Besides, there may be a correlation between the occurrence of pain and the amount of limb lengthening, indicating that the likelihood of experiencing pain increases with a greater amount of limb lengthening. Therefore, pain management should be prepared for patients with limb lengthening exceeding the critical size length [i.e., when the length is 2–2.5 times greater than the diameter of the affected long bone (26)].

External fixation time of the limb lengthening usually requires a longer time compared to bone transport for repairing bone defects, which increases the difficulty of postoperative management and the risk of complications (7). In this cohort, pin tract infection occurred in 4 patients. While pin tract infection can be effectively managed, multiple causes result in it, including thermal injury from intraoperative Kirschner wire penetration, thick soft tissue around the Kirschner wire, and irritation from the procedure of distraction. Hence, the precise intraoperative technique is crucial, along with timely adjustments to the distraction rate based on the pressure between the fixation Kirschner wire and the surrounding soft tissue during the distraction phase, which may reduce the risk of pain while minimizing pin tract reactions.

Long external fixation time also poses a risk for adjacent joint issues, with a particularly high incidence of clubfoot deformity (8). To prevent and correct foot drop deformity, we recommend the installation of a U-ring on the foot. Despite this, two patients developed foot drop deformity after removal of the external fixator, which was successfully treated with tenodesis. Additionally, regular functional exercises of the knee during limb lengthening are essential for maintaining the range of motion of the knee. The cumbersome appearance of the external fixator can significantly impact the patient's quality of life, demanding higher levels of patient compliance. Consequently, this technique is not recommended for patients with mental disorders who may struggle to adhere to this treatment period. ankle fusion (or any joint-related procedures) may influence the functional outcomes but would not affect the Paley score in terms of alignment and healing.

There were several potential limitations in this study. Firstly, this study was conducted retrospectively with a small sample size. Secondly, there is no unified algorithm for the management of limb shortening deformity after replantation of limb amputation. A gait assessment could be performed in future research to determine whether a replanted and relocated limb is functionally similar to a prosthetic leg. Thirdly, there is a lack of comparison with the bone and functional results of other treatment methods. Thus, a prospective multi-center study with a large sample size is still crucial for the clinical application of limb lengthening.

## Conclusion

Limb lengthening based on the Ilizarov technique was a safe and effective method for the treatment of shortening following successful replantation of traumatic lower leg amputation, and it could yield satisfactory postoperative bone and functional results. However, limb lengthening should be initiated at least 6 weeks after the successful replantation. The long treatment period usually associated with this method increases the risk of complications, necessitating good patient compliance with meticulous postoperative management and follow-up guidance to minimize these risks.

## Data Availability

The original contributions presented in the study are included in the article/Supplementary Material, further inquiries can be directed to the corresponding authors.
